# Barriers and facilitators of childhood flu vaccination: the views of parents in North East England

**DOI:** 10.1007/s10389-022-01695-2

**Published:** 2022-02-18

**Authors:** Timothy Price, Elaine McColl, Shelina Visram

**Affiliations:** 1 Newcastle University, 155 Percy Street, Apartment 52 The Bruce Building, Newcastle Upon Tyne, NE1 7RP UK; 2 Population Health Sciences Institute, Newcastle University, Newcastle Upon Tyne, UK

**Keywords:** Influenza, Vaccine, Pre-school children, Vaccine hesitancy, Parental, Qualitative

## Abstract

**Aim:**

The aim of this study was to complete a descriptive qualitative investigation of parents’ perceptions of the barriers and facilitators to flu vaccination for pre-school children.

**Subject and method:**

Participants were recruited through various communication channels to maximize sample variation. Invitations to participate in the study were sent to members of the Newcastle University Parent Network and to parents who had participated in previous research conducted at Newcastle University. Twelve participants (six with vaccinated children, six whose children were not vaccinated) took part in a semi-structured interview via Zoom. Transcripts were coded using Nvivo 12 and data were thematically analyzed using the COM-B model of health behavior change.

**Results:**

Participants whose children were not vaccinated against flu nonetheless generally held favourable views of vaccination and reported low concern about side-effects. Barriers involved a combination of internal and external factors, mainly a lack of convenient access to vaccination opportunities and flu vaccination being a low priority for busy parents.

**Conclusion:**

Our findings suggest that socioeconomic status, which is known to influence other vaccination behaviors, may influence uptake of the flu vaccine in this population. Inconvenient vaccination opportunities and a lack of awareness of the need to vaccinate are major barriers to uptake for some parents. The finding that belief that flu vaccination is a civic responsibility is a new contribution to the literature.

**Supplementary Information:**

The online version contains supplementary material available at 10.1007/s10389-022-01695-2.

## Introduction

Influenza, commonly known as “the flu,” is an acute respiratory infection caused by influenza viruses. Most people who are infected with influenza recover within a week and do not use health services; however, those who do can cause significant burden to primary and secondary care (Fleming et al. 2016; Uwemedimo et al. 2012). Populations at high risk for influenza-related hospitalization and death include pregnant women, children between 6 months and 5 years, and adults aged 65 and older (Ghebrehewet et al. 2016).

In the UK, the impact on the healthcare system tends to be concentrated from late January through early March (Fleming et al. 2016). During the average UK flu season, there are an estimated 857,996 visits to primary care facilities as a result of the virus (Fleming et al. 2016). Children between 2 and 5 years old with influenza are more likely than adults to utilize primary care services (Fleming et al. 2016), while those under 2 are hospitalized due to influenza at a similar rate to adults over 65 (Poehling et al. 2006). Young children who are not vaccinated are more likely to contract flu at nursery or school and to act as “super-spreaders” who transmit the virus at a rapid rate (NHS England 2017).

Vaccination is the single most effective way to protect an individual from contracting influenza and to prevent widespread transmission of the virus (Ghebrehewet et al. 2016). Barriers to childhood flu vaccination identified in previous studies include: a perceived lack of need for the vaccine (Gazmararian et al. 2010; King and Leask 2017; Paterson et al. 2018a, b; Sampson et al. 2011); fear of severe adverse reactions (Gnanasekaran et al. 2006; Goss et al. 2020; Lau et al. 2013; Offutt-Powell et al. 2014; Paterson et al. 2018a, b; Sampson et al. 2011); and lack of convenient access (Daley et al. 2006; Gazmararian et al. 2010; Goss et al. 2020; Lind et al. 2015; O’Leary et al. 2015; Uwemedimo et al. 2012). The belief that the vaccine effectively protects a child from flu (Bhat-Schelbert et al. 2012; Biezen et al. 2018; Daley et al. 2006; Gnanasekaran et al. 2006; Paterson et al. 2018b) and a doctor’s recommendation (Daley et al. 2006; Gnanasekaran et al. 2006; Lau et al. 2013; O’Leary et al. 2015; Offutt-Powell et al. 2014) are common facilitators. To date, no published research has qualitatively explored barriers and facilitators of vaccine uptake for pre-school children in England, where flu vaccination rates tend to be sub-optimal. During the 2019/2020 flu season, the NHS targeted 50% flu vaccination uptake in pre-school-aged children, but achieved only 43.4% uptake (Public Health England, 2020b).

The aim of this study was to investigate the barriers and facilitators to flu vaccination for pre-school children in the North East of England. This area was an ideal research setting, given the significant variance in vaccination uptake rates by locality (Public Health England 2020a). The region has high levels of routine childhood vaccination uptake, making the below-average flu vaccination rates worthy of investigation (Public Health England 2020b).

## Methods

The study utilized an exploratory qualitative research design (Smith et al. 2011), which is appropriate for gaining insights into phenomena that are not yet well understood (Kim et al. 2017). Recruitment involved a variety of approaches to maximise sample variation, while also considering constraints imposed by COVID-19. An invitation email was sent to the Newcastle University Parents’ Network (NUPN) and to participants from past studies who had consented to be contacted with regard to future research. The study was also advertised in the NUPN newsletter, via social media, and through interested parents sharing the invitation with friends and colleagues.

Parents and guardians were eligible to participate in the study if they cared for at least one child aged 2 or 3 years old and eligible to receive the vaccine. Recruitment took place between May and July 2020, when data saturation, the point when generating additional data does not yield additional themes, was reached (Fusch and Ness 2015). The authors established that data saturation had been reached by discussing the themes generated during analysis and reaching a consensus.

Potential participants completed an online expression of interest form which collected demographic information (parent’s and child’s ages, postcode, employment status) and contact details. Purposive sampling, a form of non-probability sampling in which researchers select eligible individuals to participate in a study, was used to maximise variation in children’s vaccination status, parents’ employment status, and parental gender (Lavrakas 2008). Of the 24 people who completed the form, 16 were invited to take part in the study, 12 of whom responded to the invitation and completed an interview. Table 1 gives an overview of the sample.

**Table 1:** Participant demographic information

Participant	Relationship to child	Age	Number of children (of any age)	Age of pre-school children	Pre-school child(ren) vaccinated in 2019/20	Employment status	2019 Index of Multiple Deprivation* decile
Participant 01	Mother	32	2	2	No	Not employed	8
Participant 02	Mother	33	1	3	No	Part time	3
Participant 03	Mother	36	1	3	Yes	Full time	10
Participant 04	Mother	38	2	2	No	Part time	6
Participant 05	Mother	49	2	3, 4	Yes	Full time	9
Participant 06	Mother	32	2	2	No	Part time	5
Participant 07	Mother	41	1	3	Yes	Full time	10
Participant 08	Mother	32	1	2	No	Part time	4
Participant 09	Father	39	4	4, 4 (twins)	Yes	Full time	9
Participant 10	Mother	35	2	2	No	Not employed	9
Participant 11	Mother	42	2	3	Yes	Full time	10
Participant 12	Father	38	2	2, 4	Yes	Full time	7

^*^ IMD is a measure of deprivation by by LSOA. Higher deciles equate to less deprivation. For example, a participant with an IMD decile of 10 lives in a LSOA that is among the 10% least deprived in England (‘English indices of deprivation 2019’)

Participants were provided with an information sheet describing the purpose of the study and how their data would be used. They were also given an electronic copy of the consent form, which was completed verbally at the start of each interview. After the interviews, participants were sent a debrief sheet and a £10.00 e-gift card to thank them for their time. Approval for the study was obtained from the Newcastle University Faculty of Medical Sciences Research Ethics Committee (ref. 1901/1882).

Data were collected through semi-structured interviews, using a topic guide developed by the research team based on a review of existing literature. The topic guide was designed to investigate parental perceptions of influenza, views on the vaccine, and the decision-making process in having a child vaccinated. Due to the COVID-19 pandemic, all interviews were conducted remotely and audio-recorded using the online conferencing service Zoom. The Zoom recording feature creates a transcript of the meeting; these transcripts were checked to improve accuracy and ensure anonymity.

Data were coded using the qualitative analysis software NVIVO 12 Pro. Codes were formulated inductively by assigning labels to data extracts and assigning them to free nodes, which were then grouped into tree nodes and used to generate emerging themes. A second team member coded a selection of transcripts to validate the codes.

**Fig. 1:**
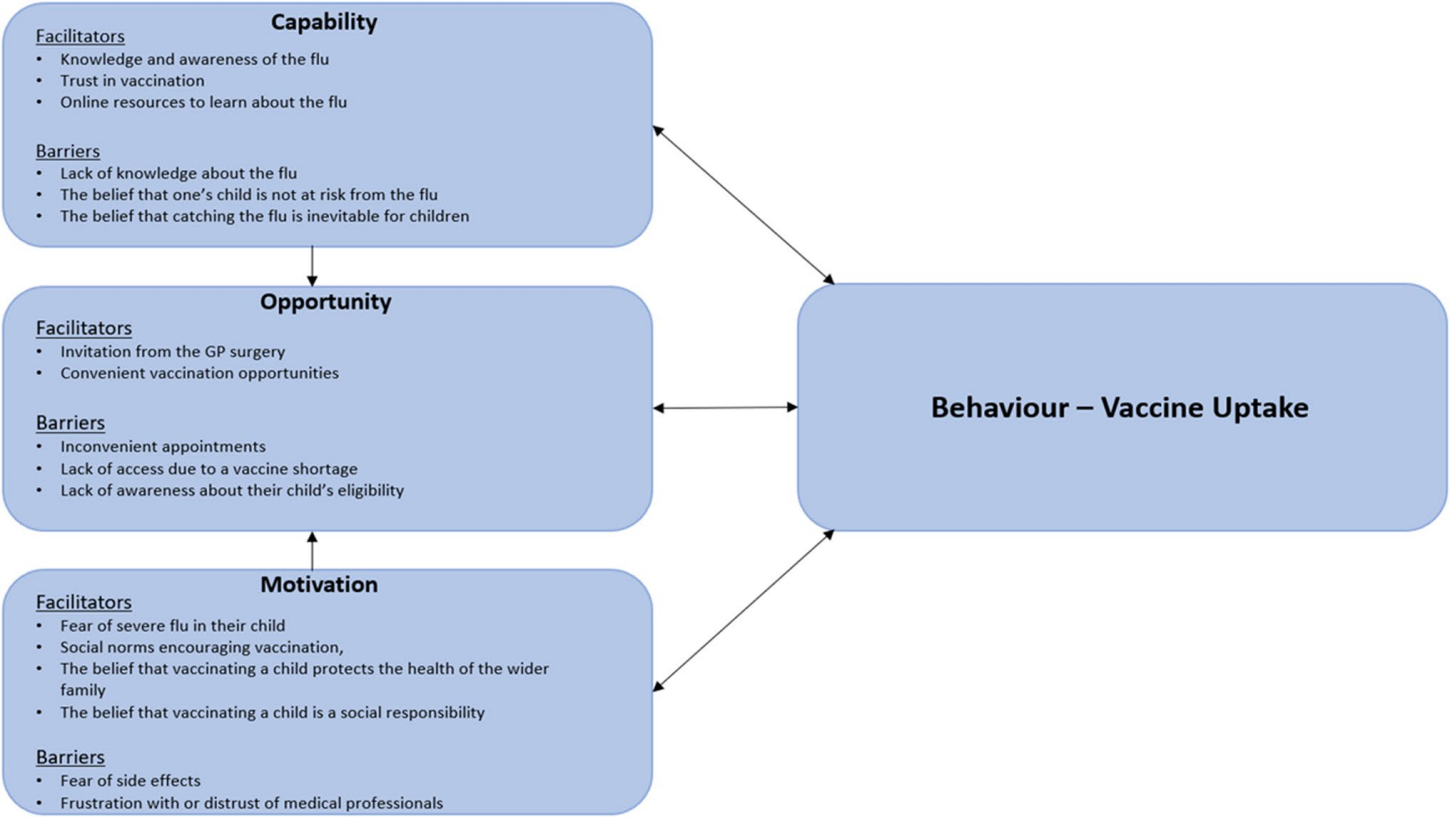
Principal findings mapped onto the COM-B framework. Framework developed by Michie et al. (2011)

Emerging themes were further organised under the three headings of the COM-B model of health behaviour change: capacity, opportunity, and motivation (Michie et al. 2011). The COM-B model explains that, to engage in a health behaviour, individuals need to have the *capability* to take an action, an appropriate *opportunity* and sufficient *motivation* to do so (Michie et al. 2011). Capability refers to a person’s psychological and physical ability to take an action, including their knowledge of the need to do so and their ability to develop that knowledge. Opportunity refers to all of the forces external to an individual that prompt an action or make it possible. Motivation refers to the processes that drive decision-making, including emotional responses, social pressures, and deliberative decision-making. COM-B is an established framework for understanding how individuals initiate health behaviours, and has been used in a previous study relating to flu vaccination uptake in pre-school children (Biezen et al. 2018; Michie et al. 2011). Figure 1 presents the study findings mapped onto the COM-B framework (Michie et al. 2011).

## Results

Twelve parents participated in an interview, six of whom had children who had been vaccinated for flu in the last year. Parents’ whose preschool children were vaccinated tended to be older than those whose children were not vaccinated (median age 40 vs 32.5 years). They were also likely to live in less-deprived areas and all were in full-time employment, whereas those whose pre-school children were not vaccinated were either in part-time employment or not employed.

### Capability — facilitators

Three of the identified facilitators of pre-school flu vaccination fit this category: knowledge and awareness of the flu, trust in vaccination, and the NHS website and other online resources.

Participants were aware of influenza and could accurately describe its symptoms in young children. Many understood that colds and flu were not the same thing, correctly sharing the belief that the flu was a more severe illness than a common cold.

Some were aware that flu is a seasonal illness and that their children were more likely to be exposed to it if they attended nursery or spent time around other children. Others were confident that they could identify flu symptoms in their child and would know how to respond. Some reported the belief that flu is a serious illness for children and that they would be concerned if their child were to catch it.

*“I think flu is quite a quite severe illness really. So it’s not something that I would take lightly.” *

*–Participant 07*

*“There is very, very, very little risk in giving them the vaccine. It’s well-proven, well-serving vaccine. And rather than them getting ill, and it might be mild, you know, you never know.” *

*–Participant 09*

The flu vaccine was perceived to be safe and to effectively reduce the risk of flu. Since the vaccine had been available for a long time, participants believed that it must be well understood, and that the NHS would not recommend it if it were not safe.

The NHS website (https://www.nhs.uk/conditions/) was used to learn about how flu affects children and about the vaccine itself; participants trusted the information on the website and believed it to be an authoritative source.

Other online resources were also used to learn about the flu, but only if participants felt the source was credible. Examples included the BBC, the US Centers for Disease Control and Prevention, the World Health Organization, and the Mayo Clinic. Parents were often cautious about misinformation and did not view online forums or social media as credible sources.

### Capability — barriers

Three barriers are included under the capability heading: lack of knowledge about the flu, the belief that one’s child is not at risk from the flu, and the belief that catching flu is inevitable for children. While many participants had sufficient knowledge and awareness of the flu, two felt that they lacked this knowledge and were not aware of how flu could affect children.

*“To be honest, I’ve never really thought about children getting flu and how, how badly they would get it… But I don’t know about in terms of children, I don’t know if children can get proper flu.”*

*– Participant 05*

*“I think just a regular flu is something that we’re all going to come into contact with, with it in our daily lives. I think that I can’t really protect him from it, it’s something that he’s going to get.”*

*– Participant 02*

Others shared the belief that their children were generally healthy and did not feel the need to worry about them having a severe case of flu. While these parents made it clear that they did not want their child to catch the flu, they would not have a high level of concern if they did.

Some believed their child would inevitably catch flu and that there was little that could be done to prevent it. These participants viewed flu as a common childhood illness that did not warrant particular concern.

### Opportunity — facilitators

Two facilitators are included under this heading: invitation from the GP surgery and convenient vaccination opportunities.

Participants received a letter from their GP surgery informing them of their child’s eligibility to receive the flu vaccine, which generally prompted them to make an appointment to have their child vaccinated. For some, the letter reminded them that flu vaccine season was starting. For others, it was the first notice a parent received that their child was eligible.

*“I think the letter served as a bit of a reminder, and I may have forgotten or not got round to it as quickly if I didn’t get the letter as a reminder.” *

*– Participant 12*

Providing ample access to vaccination opportunities is a key facilitator to encouraging uptake, particularly for parents of young children. Participants commonly described the process as ‘straightforward’.

*“It was a dead quick and straightforward process. Sat in the waiting room and then going along and having the appointment.” *

*– Participant 12*

### Opportunity — barriers

Three barriers related to opportunity were identified: inconvenient appointments, lack of access due to a vaccine shortage, and lack of awareness about their child’s eligibility.

For some, vaccination appointments were scheduled at inconvenient times, and parents were not able to attend or needed to reschedule.

*“it was quite short window of clinics that they had available and they only were available at certain times on Friday. Now, I work part-time but Friday’s one of the days that I’m in work. And also my daughter’s in playgroup that day. So it was, so it’s more a logistic problem that we couldn’t get there because it was on a day that I work.” *

*–Participant 06*

Some participants had difficulty having their child vaccinated because of a vaccine shortage. They were told either that their GP surgery had no vaccine available or that the vaccine was being reserved for high-risk groups, which led to frustration.

One participant was not aware that her child was eligible. She did not receive a notice from her GP surgery, and did not think to ask whether her child should receive the vaccine. Another had recently moved and did not know how to have her daughter vaccinated because she had not yet registered with a GP.

*“I was looking at paying for it privately, but nobody would do the nasal thing [spray] privately … I then rung up other pharmacies locally and so "Will you do it privately for child?" And most said “no”, they wouldn’t. I found it very difficult to find anyone that would give it to a 3-year-old.” *

*– Participant 11*

*“We weren’t offered it. I don’t know whether… I’m not entirely sure why we haven’t. But I certainly didn’t explore it either … I’m not sure, but honestly, it didn’t actually occur to me to investigate.”*

*– Participant 04*

### Motivation — facilitators

Four facilitators are included under this heading: fear of severe flu in their child, social norms encouraging vaccination, the belief that vaccinating a child protects the wider family, and the belief that vaccinating a child is a social responsibility.

Participants feared that their child would have a severe case of flu if they were to get sick. Two had a child with a health condition that may put them in a high-risk group for complications from the flu.

*“In my household definitely my daughter’s got, she’s suffered from viral wheeze. So, she’s been hospitalized a few times as a result of that. So, we take it quite seriously … I do get concerned about my kids getting the flu and particularly if my daughter got it ‘cause I know all her, she’s not got a great immune system.” *

*– Participant 06*

Others were also concerned that their child may develop a severe infection despite not being in a high-risk category.

Positive social norms surrounding vaccination were expressed in a variety of ways. Some participants indicated that vaccination was the ‘normal’ thing to do. Others felt that accepting the vaccine was a foregone conclusion.

*“So even if I read something which made me think "is this absolutely necessary?" I think, as a parent, if you didn’t do it [have your child vaccinated for flu], I would never forgive myself if something happened.” *

*– Participant 04*

*“So my mum’s, my dad’s 83, my mum’s 73 and they’re both, you know, fit and well. But, yeah, I think I was worrying for them specifically as well that if someone was to bring flu to them, that wouldn’t be a good thing.” *

*– Participant 03*

*“I guess I’ve just accepted that it’s just a good, sensible thing to allow them to have done. I think it’s been accepted that it’s one of those things that it’s just offered and that you just got without complaints.” *

*– Participant 10*

There was a belief that having a child vaccinated against flu extends protection to the family as a whole. Some valued this collective protection because it helped keep vulnerable family members, such as grandparents, safe from infection.

Others valued this protection because of the frustration and inconvenience associated with having an illness spread through the household.

*“My children cope better with being ill than we do, as parents, so I wouldn’t actually be that concerned and actually selfishly, the concern becomes that we are going to get ill … And parenting as a poorly parent is no fun at all, you know?” *

*– Participant 04*

Concern that their child would spread the flu to someone outside the family in a high-risk group also motivated parents to have their child vaccinated. Finally, some expressed the belief that it was a civic responsibility of those who could be vaccinated to receive the vaccine to contribute to herd immunity.

### Motivation — barriers

Two barriers are included under the motivation heading: the fear of side-effects and frustration with or distrust of medical professionals.

There were some general concerns about the side-effects of the vaccine and how they would impact their children. Participants worried that the vaccine would cause their child to be tired, have a fever, and be temporarily more susceptible to other illnesses. Explanations for these concerns included hearing from another parent that the vaccine had negative side-effects, and concerns about being unwell during a holiday.

*“I was concerned about having a negative effect on his health for the holiday. I understand that a vaccine can’t give you the flu, it won’t make you ill, but I was concerned about him, if his immune system was to be busy off reacting to the vaccine in the right ways, he might come down with something else, like a secondary thing.” *

*–* *Participant 02*

Some participants felt frustrated with their GP and were skeptical of the NHS. While these feelings did not prevent them from having their child vaccinated, their concerns may have impacted their motivation to vaccinate.

## Discussion

Participants in this study generally held favourable views of vaccination and reported low concern about side-effects.’Anti-vaxx’ views were not identified as a major barrier to flu vaccination for pre-school children. Participants who did not vaccinate their children were usually prevented from doing so through a combination of internal and external forces, mainly a lack of convenient access and flu vaccination being a low priority for busy parents. Parents who did not have convenient access to vaccination through their GP often went without doing so, despite indicating that they would prefer to have had their child vaccinated. Contrary to what might be expected, participants who worked full-time appeared to have greater flexibility in their working schedule than those who worked part-time. Inconvenient appointment and vaccination clinic times were a greater barrier to part-time workers; these parents tended to live in more deprived neighborhoods and may have worked in jobs with stricter shift schedules. Parents whose children were vaccinated reported wanting to protect older family members and other vulnerable individuals from contracting the virus.

Public discourse generally attributes failure to vaccinate children to anti-vaxx views or to parental ignorance and apathy (Benecke and Deyoung 2019; Hussain et al. 2018). Vaccine hesitancy, when individuals delay or refuse vaccination despite the availability of vaccines, is a matter of growing concern, particularly as a result of the COVID-19 pandemic (Dror et al. 2020). The Vaccine Hesitancy Matrix proposes that vaccine hesitancy has three components which can serve as barriers to vaccination; complacency, confidence, and convenience (Macdonald 2015). *Complacency* refers to the perceived belief that the risk of vaccine preventable illness is low; *confidence* is trust in the safety and effectiveness of vaccines and the systems that deliver them; *convenience* is the physical availability and geographical accessibility of vaccination opportunities (Macdonald 2015). Past studies on barriers to flu vaccination for pre-school children identified that parents are concerned about their child having a severe adverse reaction, and that these fears can prevent a parent from having their child vaccinated (Gnanasekaran et al. 2006; Goss et al. 2020; Lau et al. 2013; Offutt-Powell et al. 2014; Paterson et al. 2018a, b; Sampson et al. 2011). A study conducted in the US in the early 2000s identified positive social norms surrounding flu vaccination as a facilitator of uptake for pre-school children (Daley et al. 2006). While there is limited literature on the facilitators of flu vaccination in this population, evidence from studies involving other age groups or other childhood vaccinations is relevant. The introduction of the in-school vaccination programme successfully increased uptake of the flu vaccine in school-age children in England (Moulsdale et al. 2017; Paterson et al. 2018b). Pharmacies have been effective venues through which to promote uptake of the flu vaccine in adults (Burson et al. 2016). In England, community pharmacies may be an ideal setting to promote vaccination because they tend to be more accessible in areas of high deprivation than other health services (Todd et al. 2018).

Our findings suggest that socioeconomic status, which is known to influence other vaccination behaviors, may influence uptake of the flu vaccine in this population. Inconvenient vaccination opportunities were identified as a major barrier to uptake. The belief that having a child receive the flu vaccination is a civic responsibility is a new finding that this study contributes to existing knowledge on this topic. The study also provides additional evidence that vaccination being perceived as a social norm facilitates uptake, which has only been documented in one previous study (Daley et al. 2006).

The COM-B model of health behavior change advances a nuanced approach to barriers and facilitators. When deciding whether or not to initiate a health behavior, individuals often face both barriers and facilitators (Michie et al. 2011). In the context of this study, the presence of a barrier did not necessarily mean that a parent would not vaccinate their child, nor did the presence of a facilitator guarantee that a child would be vaccinated. Instead, it is ultimately a combination of factors that determine whether a child is vaccinated. This suggests that the public discourse surrounding failure to vaccinate may be oversimplified, and that a more nuanced view, such as the one advanced by the Vaccine Hesitancy Matrix, is warranted (Macdonald 2015). Parents whose children were not vaccinated were generally well informed of the flu and the risk that it posed to their child, believed the vaccine to be safe and effective, and were willing to have their child vaccinated if they had a convenient opportunity to do so.

This study involved a relatively small sample of parents from one English region and the findings may not be generalizable to other contexts. We did not collect information on participants’ ethnic background, so it is not clear whether our findings include the perspective of those from minority ethnic backgrounds. The recruitment methods provided access to a population with links to Newcastle University, who were more likely to be in employment and therefore of higher socio-economic status. Additionally, several participants had some degree of familiarity with health research, from past experience or professional training. Despite these limitations, we achieved a relatively diverse sample, which allowed us to corroborate existing evidence regarding the relationship between socio-economic status and vaccine uptake. Additionally, the use of the COM-B framework highlights opportunities for interventions to improve uptake in this population.

## Conclusion

Future research examining barriers and facilitators of flu vaccination should investigate the role that social norms and civic responsibility play in facilitating uptake. Policymakers should note the importance of convenient vaccination opportunities as a facilitator of vaccination for pre-school children and should seek to increase avenues for vaccination. The provision of opportunities to have pre-school children vaccinated at community pharmacies or in nurseries could be especially effective in reaching parents who wish to have their children vaccinated late in the season or those whose work schedules do not align with the clinics offered by their GP surgery.

## Supplementary Information

Below is the link to the electronic supplementary material.

Supplementary file1 (DOCX 12 KB)

## Data Availability

The data underlying this article were extracted from anonomyised transcripts of participant interviews, which are available at Newcastle University’s data repository: https://doi.org/10.25405/data.ncl.14242040.
