# Supplementary material for: Barriers and facilitators of childhood flu vaccination: the views of parents in North East England
Source: Z Gesundh Wiss. 2022 Feb 18;30(11):2619–26. doi: 10.1007/s10389-022-01695-2 (PMC8853737; doi:10.1007/s10389-022-01695-2)
Supplement: Supplementary file 1 — Supplementary file1 (DOCX 12 KB) [file 10389_2022_1695_MOESM1_ESM.docx]

The data underlying the analysis and conclusions of this article were extracted from anonomyised transcripts of participant interviews, which are available at Newcastle University’s data repository: [10.25405/data.ncl.14242040](https://doi.org/10.25405/data.ncl.14242040)
